# Investigating Health Disparities in Online Pediatric Skull Fracture Information: An Analysis of Resource Quality and Readability

**DOI:** 10.7759/cureus.114029

**Published:** 2026-08-05

**Authors:** Landon E Ebbert, Tyson Pace, Cameron Wilkinson, Timothy O'Daniel, Cody Messick, Joshua L Ebbert, Jeffrey Mecham, David Crockett

**Affiliations:** 1 Otolaryngology - Head and Neck Surgery, Mayo Clinic Alix School of Medicine, Phoenix, USA; 2 Physics and Astronomy, Brigham Young University, Provo, USA; 3 Otolaryngology, Phoenix Children’s Hospital, Phoenix, USA

**Keywords:** health-care equity, pediatric, quality, readability, skull fracture

## Abstract

Introduction

Families often seek medical information online; this study assesses the accessibility of online pediatric skull fracture materials by evaluating the quality and readability of easily obtained sources.

Methods

Six terms were queried using Google (Google LLC, Mountain View, California, United States), Bing (Microsoft Corporation, Redmond, Washington, United States), and Yahoo (Yahoo Inc., New York City, New York, United States). Twenty-four unique websites were categorized as commercial, academic, or medical practice. Readability was assessed using Flesch Reading Ease Score (FRES) and Flesch-Kincaid Grade Level (FKGL), while quality was measured using Quality Evaluation Scoring Tool (QUEST) and the DISCERN tool. Analysis of variance (ANOVA) compared readability and quality scores by authorship classification. Linear regression was performed for QUEST vs DISCERN scores and quality vs readability.

Results

Among 24 unique sites, 8 (33%) were commercial, 10 (42%) from academic institutions, and 6 (25%) from medical practices. Mean FRES and FKGL scores corresponded to an eighth-grade reading level. Mean DISCERN and QUEST scores were 48.8 ± 8.7 (Fair) and 15.0 ± 6.3 (Fair). ANOVA indicated no significant difference in readability scores by website type (p > 0.05). Linear regression revealed a significant correlation between QUEST and DISCERN scores (p = 0.011) but no significant correlation between readability and quality metrics (p = 0.31).

Conclusions

Online materials do not meet the American Medical Association’s recommended sixth-grade level. Sites lacked proper source attribution and clear explanations of treatment options. QUEST and DISCERN both measure quality in comparable ways. Readability and quality were not significantly linked. Complex language, reflected in high readability scores, may hinder health literacy, especially among lower socioeconomic status populations. Efforts should focus on improving content quality while simplifying language to promote health equity.

## Introduction

Skull fractures represent a common injury among pediatric patients, affecting 2%-20% of all children presenting to outpatient physicians for head trauma [1]. Appropriate and timely evaluation of such pediatric head injuries is crucial due to the potential for underlying intracranial injuries and the need for proper management [2]. Serious complications may include intracranial hemorrhage, cranial nerve injury, cerebrospinal fluid leak, and hearing loss. Injuries can be complicated and require involvement of multiple pediatric subspecialties, including craniofacial surgery, neurosurgery, otolaryngology, audiology, and speech and rehabilitative services. When a child experiences a head injury and may have a skull fracture, their guardian often wants to understand the child’s symptoms and know when it is appropriate to seek medical evaluation from a physician.

The internet is one of the most accessible and commonly used patient-facing resources for gathering health information. Due to the plethora of information easily accessible to patients via the internet, there has been significant concern expressed regarding the quality and credibility of such materials [3]. Unbiased, well-written, and nonproprietary information will aid patients in the decision-making process, potentially improving patient outcome expectations and increasing treatment compliance, yet many websites do not adhere to established quality and credibility standards [4]. Several tools have been created for the assessment of quality, transparency, and validity of online patient information. Poor quality of medical patient-facing literature is not the only challenge; frequently, such online information is written with literary complexity and jargon that surpasses the reading level of the patients who read it, leading to misinterpretation and inappropriate self-diagnosis or treatment [5].

In this study, we aimed to better understand the quality of materials for pediatric skull fractures patients by using both DISCERN [6] and the Quality Evaluation Scoring Tool (QUEST) [7], two metrics that have been used in past studies for similar quality evaluation purposes [8]. Additionally, readability scores of the websites were analyzed to further provide insight into the practical benefit of information patients likely read. To the best of our knowledge, this is the first time such quality and readability analyses have been performed on online patient information for pediatric skull fractures. While quality and readability studies have been performed in the past on various healthcare topics [9], few if any have employed both DISCERN and QUEST. Thus, this study serves additionally as a rigorous, systematic comparison of the two methods.

## Materials and methods

Website selection and category assignment

On March 20, 2024, a Google Chrome (Google LLC, Mountain View, California, United States) browser in incognito mode with no browsing history in Phoenix, Arizona, was used to search the six terms: “pediatric skull fracture,” “child skull fracture,” “pediatric head fracture,” “child head fracture,” “pediatric cranial fracture,” and “child cranial fracture.” Each term was searched separately using Google (Google LLC, Mountain View, California, United States), Bing (Microsoft Corporation, Redmond, Washington, United States), and Yahoo (Yahoo Inc., New York City, New York, United States), and the top 20 results of each search were recorded. These three search engines were employed since they are three of the most used search engines by English speakers [10].

Only search results that yielded English websites from the general results section were extracted. Sponsored posts, featured snippets, video-based, image-based, message-boards, and “related searches” were all excluded. Search results that did not pertain to pediatric skull fractures, produced security warnings, contained a broken link, led to PDF or file instead of website, or were located behind paywalls were further excluded. Academic or websites that were clinician-oriented and were beyond the scope of patient materials were likewise excluded as patients would likely not read them. Duplicate sites were removed during the screening process. A panel of two reviewers participated and jointly performed the application of the exclusion and inclusion criteria.

Once the websites to be included in the study were determined, each source was categorized as academic, commercial, government, medical practice, single surgeon, or social media. Websites were categorized into the following groups: academic (affiliated with medical university centers, universities, or professional societies), commercial (organizations providing medical information without delivering medical care), medical practice (healthcare organizations without academic affiliations), single surgeon (individual physicians without academic ties), and social media (community-driven platforms providing collective input), as has been done in other studies [11].

Readability analysis

Prior to readability score calculation, text from each website was copied and pasted as plain text into a Microsoft Word (Microsoft Corporation, Redmond, Washington, United States) document. Editing, as has been suggested by previous publications, was conducted to ensure accurate readability scores; such edits included removing decimals, colons, periods in abbreviations, dashes, and semicolons [12,13]. Text not relevant to the condition such as references, menu listings, author information, and site information irrelevant to skull fractures was omitted. In the case of limitless-style websites in which scrolling down would continually load more content, only text from the first relevant article was analyzed, as has been previously done [14]. Flesch Reading Ease (FRES) [13] and Flesch-Kincaid Grade Level (FKGL) [15] readability scores were then calculated using the Editor function of Microsoft Word for Microsoft 365 MSO Version 2401 (Microsoft, Redmond, WA). An interpretation of FRES and FKGL scores can be found in Table 1. In brief, readability scores are dependent on average numbers of words per sentence and syllables per word. Higher FRES scores are associated with more accessible writing, while lower FKGL scores correlate with better readability. FRES and FKGL scores were averaged across each category of website for analysis. Standardized readability scores were calculated by averaging calculated Z-scores of both FRES and FKGL using the “STANDARDIZE” function in Microsoft Excel Version 2504 (Microsoft, Redmond, WA). These measures were selected because they are widely applied in evaluations of patient education materials, facilitating comparison with prior studies. Although additional indices such as the Simple Measure of Gobbledygook (SMOG) Index, Gunning Fog Index, Coleman-Liau Index, and Automated Readability Index are also used to assess readability, they rely on similar textual features. Therefore, FRES and FKGL were considered sufficient to characterize the readability of the included materials.

**Table 1:** Numerical scales for readability and quality scores Score interpretation criteria for FRES [13], FKGL [15], DISCERN [6], and QUEST [7]. FRES: Flesch Reading Ease Score; FKGL: Flesch-Kincaid Grade Level; QUEST: Quality Evaluation Scoring Tool.

Readability and Quality Metrics	
FRES	
0-30	College Graduate
31-50	13th-16th Grade
51-60	10th-12th Grade
61-70	8th-9th Grade
71-80	7th Grade
81-90	6th Grade
91-100	5th Grade
FKGL	
0-2	Kindergarten/Elementary
3-5	Intermediate
6-8	Middle School
9-12	High School
13-15	College
16-18	Post-graduate
DISCERN	
16-27	Very Poor
28-38	Poor
39-50	Fair
51-62	Good
63-80	Excellent
QUEST	
0-5	Very Poor
6-11	Poor
12-17	Fair
18-23	Good
24-28	Excellent

Quality analysis

DISCERN and QUEST scores were assigned using the previously set forth criteria [6,7]. Only information on the direct link from the web search was included in the review. No links were followed, and no other pages were visited when assessing the quality of the online material. Every article was reviewed independently by two reviewers, and the scores were averaged. Reviewers were blinded to the other reviewer’s scores. Scores from each reviewer were compared and significant differences (by more than one point) reconciled through unanimous decision. DISCERN and QUEST score scales can be found in Table 1 [16]. Higher DISCERN and QUEST scores indicate greater quality. Standardized quality scores were calculated by averaging calculated Z-scores of both DISCERN and QUEST using the “STANDARDIZE” function in Microsoft Excel.

Statistical analysis

Statistical analyses were performed using Microsoft Excel Version 2504 (Microsoft, Redmond, WA). Analysis of variance (ANOVA) was used to determine statistically significant differences in QUEST, DISCERN, FRES, and FKGL scores among the academic, commercial, and medical practice groups. For metrics where the ANOVA yielded significant results, post hoc two-tailed Student’s t-tests were subsequently performed to assess specific pairwise differences between groups.

To evaluate the relationship between the two quality metrics, DISCERN and QUEST, a linear regression analysis was performed. Similar regressions were performed to assess the correlation between the two readability metrics, FRES and FKGL, and to examine the relationship between readability and quality. Statistical significance for all tests was set at α = 0.05.

## Results

The website selection and screening process resulted in 24 unique websites to be included in the study, with the following categorizations, counts, and percentages: academic (n = 10, 42%), commercial (n = 8, 33%), and medical practice (n = 6, 25%) (Table 2). A complete list of the full website URLs may be viewed in Appendix 1. Of the exclusion criteria, the most frequent reasons for website exclusion included the website being behind a paywall, directed at a more technical physician audience, and duplicate representation.

**Table 2:** Queried websites with associated readability and averaged quality scores Full website URLs may be viewed in Appendix 1. FRES: Flesch Reading Ease Score; FKGL: Flesch-Kincaid Grade Level; QUEST: Quality Evaluation Scoring Tool; URMC: University of Rochester Medical Center; GOSH NHS: Great Ormond Street Hospital National Health Services; KC: Kansas City; CHOA: Children’s Healthcare of Atlanta.

Website	DISCERN	QUEST	FRES	FKGL	Website Category
GOSH NHS - Head Injuries	61	13	57.6	9.8	Academic
Stanford Children's - Head Injury	53	7	63	8.3	Academic
URMC - Head Injury	46	12	69.5	7.1	Academic
Mount Sinai - Skull Fracture	40.5	17	63.4	7.5	Academic
Stanford Children's - Head Injury in Children	48.5	11.5	71	6.9	Academic
Cleveland Clinic - Skull Fracture	63.5	25	64.7	7.4	Academic
GOSH NHS - Skull Fracture	43.5	7	80.3	5.3	Academic
Hopkins Medicine - Head Injury in Children	49	10	69.3	7.1	Academic
Saint Luke's KC - Treatment Skull Fracture	40.5	11.5	74.8	5.7	Academic
Saint Luke's KC - Understanding Skull Fracture Child	36	10	75.9	5.6	Academic
Healthline - Skull Fracture	57.5	22.5	61	8.9	Commercial
Children Birth Injuries - Skull Fractures	47.5	22.5	51	10.5	Commercial
Kids Health - Head Injury	35.5	15.5	75.8	5.8	Commercial
Medical News Today - How Serious Is a Fractured Skull?	44	18.5	61.8	8.4	Commercial
Healthline - Calvarial Fracture	63.5	26	46.3	10.3	Commercial
Cerebral Palsy Guidance - Infant Skull Fracture	55	22	58.3	9.2	Commercial
Drugs - Skull Fracture in Children	55	14	82.4	4.9	Commercial
UpToDate - Head Injury in Children and Adolescents	62.5	26	52.8	10.2	Commercial
Children's Hospital - Head or Brain Injury	51	7	59.3	9.4	Medical Practice
Nicklaus Children's - Skull Fracture	34.5	14	60.9	8.5	Medical Practice
Children's Colorado - Head Injuries	45.5	16.5	55.4	10.5	Medical Practice
Intermountain Healthcare - Skull Fracture	42.5	10	77.6	5.7	Medical Practice
CHOA - Skull Fractures	46.5	5.5	79.3	5.6	Medical Practice
Nationwide Children's - Head Injury in Children	49	16	56.8	9.5	Medical Practice

Quality

Commercial websites had the highest average QUEST and DISCERN scores at 20.9 (Good) and 52.6 (Good), respectively. Academic sites had the next highest averages scores of 12.4 (Fair) and 48.2 (Fair) on QUEST and DISCERN, respectively. Medical practice sites had the lowest average scores with 11.5 (Fair) and 44.8 (Fair) on QUEST and DISCERN, respectively.

**Figure 1:**
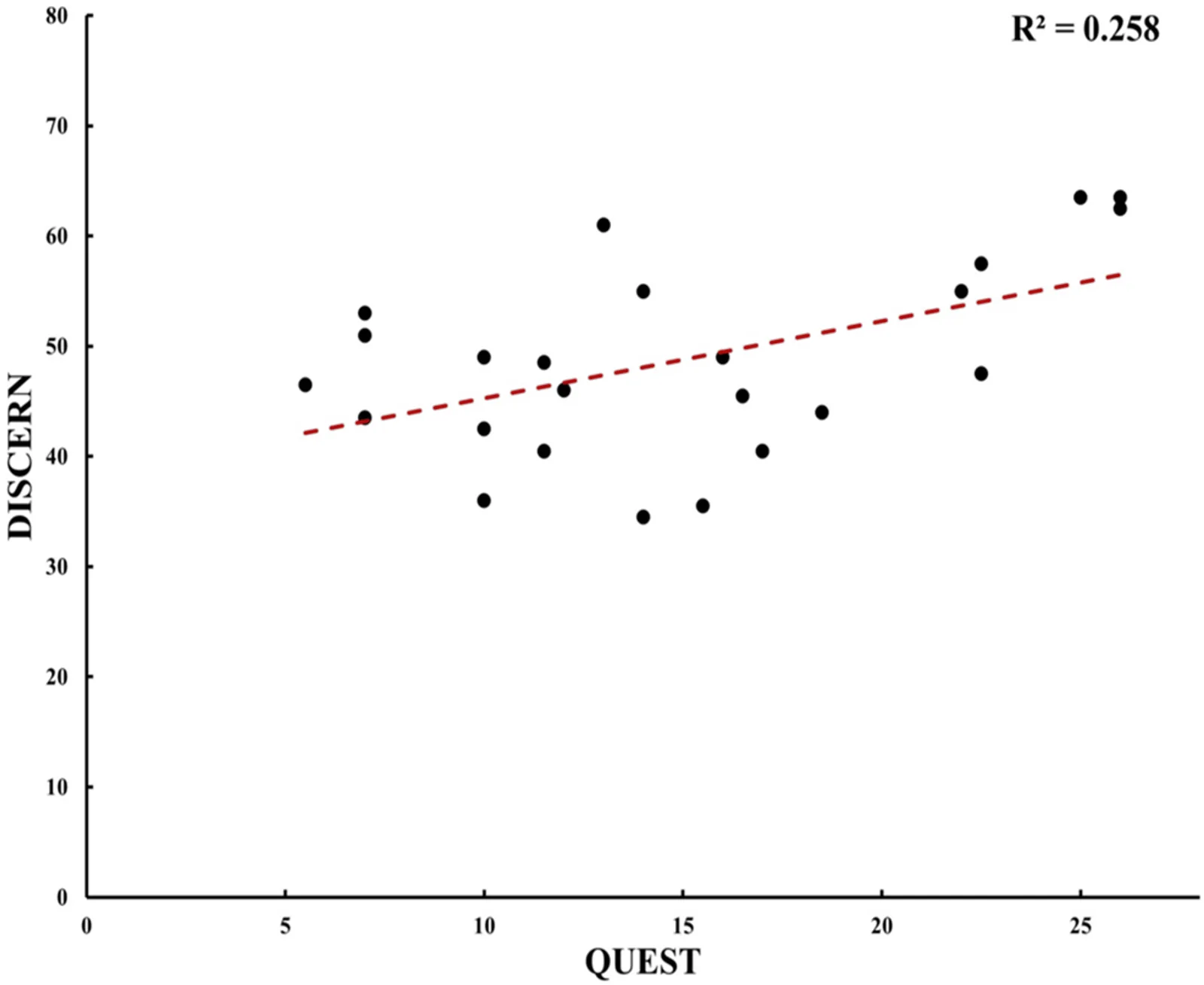
Scatter plot and linear regression analysis of QUEST versus DISCERN scores The red dashed line indicates the line of best fit (ŷ = 0.699x + 38.304; R-squared = 0.258; n = 24). QUEST: Quality Evaluation Scoring Tool.

There was a significant difference among groups’ QUEST scores (p < 0.002) but not among DISCERN scores (p > 0.05). Commercial websites exhibited significantly higher quality than both academic websites (p < 0.003) and medical practice websites (p < 0.003), but there was not a significant difference between academic websites and medical practice websites (p > 0.05). Regression analysis of QUEST versus DISCERN scores across all website categories demonstrated a significant correlation (Figure 1), yielding an F-statistic of 7.65 and a significance level of F < 0.02. While the results are significant, they show only a weak correlation (R^2^ = 0.258) between QUEST and DISCERN.

Readability

**Figure 2:**
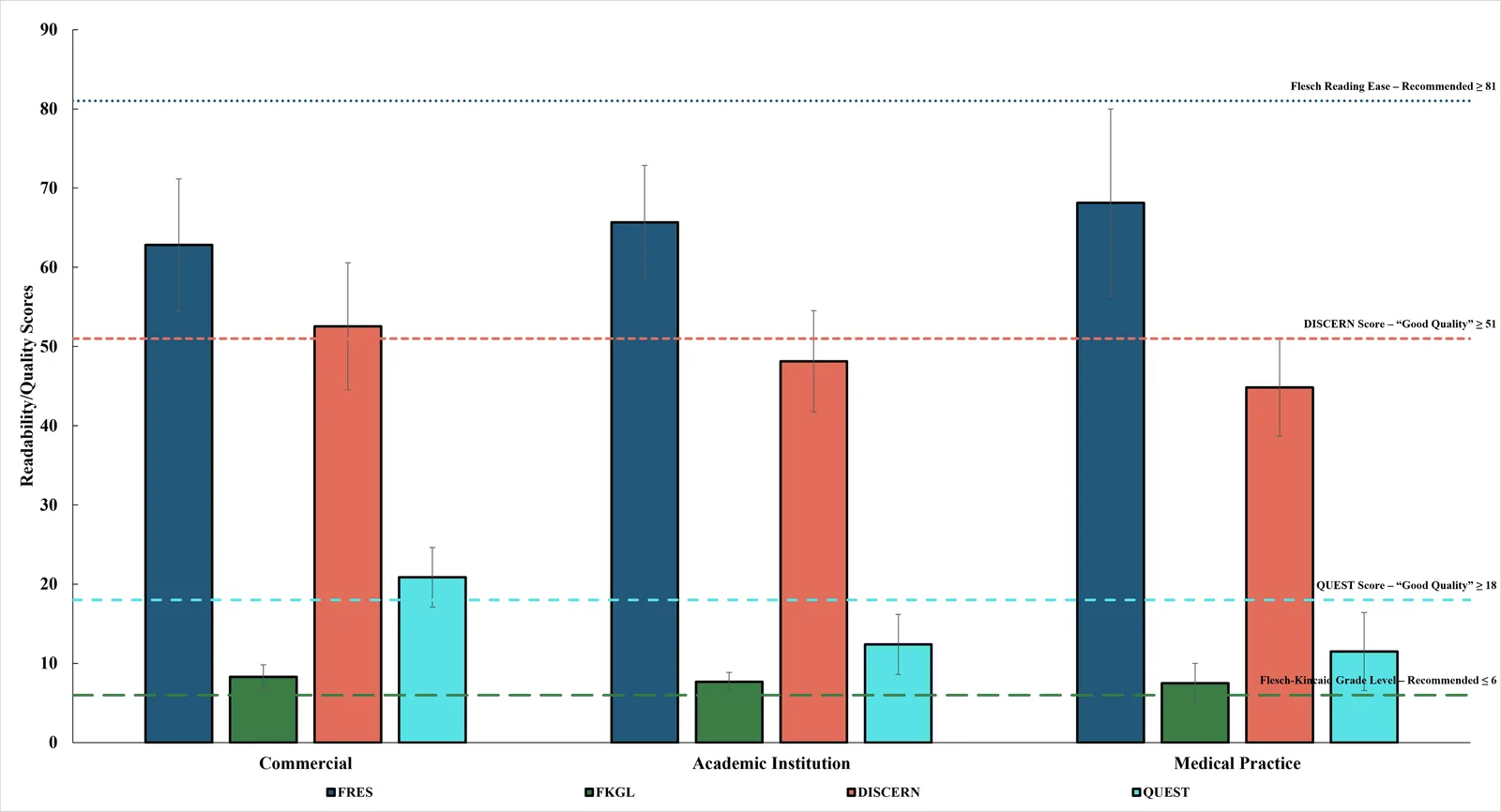
Readability and quality scores by website type Guideline targets are depicted; notably, FRES, QUEST, and DISCERN scores higher than the target line are preferred, whereas FKGL scores ideally should be below the target. Error bars represent 95% confidence intervals. FRES: Flesch Reading Ease Score; FKGL: Flesch-Kincaid Grade Level; QUEST: Quality Evaluation Scoring Tool.

**Figure 3:**
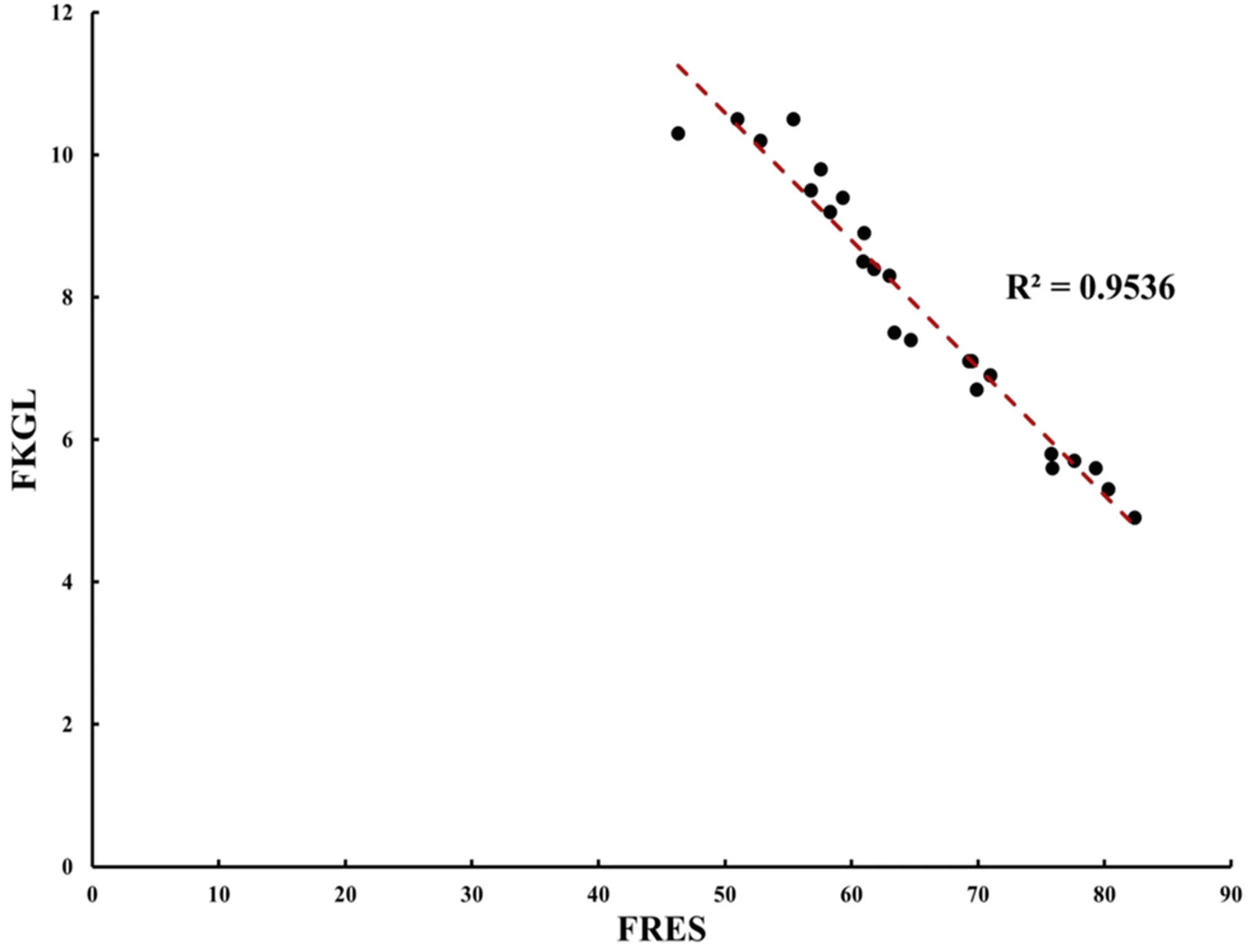
Scatter plot and linear regression analysis of FKGL versus FRES scores The red dashed line indicates the line of best fit (ŷ = 0.179x + 19.531; R-squared = 0.954; n = 24). FRES: Flesch Reading Ease Score; FKGL: Flesch-Kincaid Grade Level.

**Figure 4:**
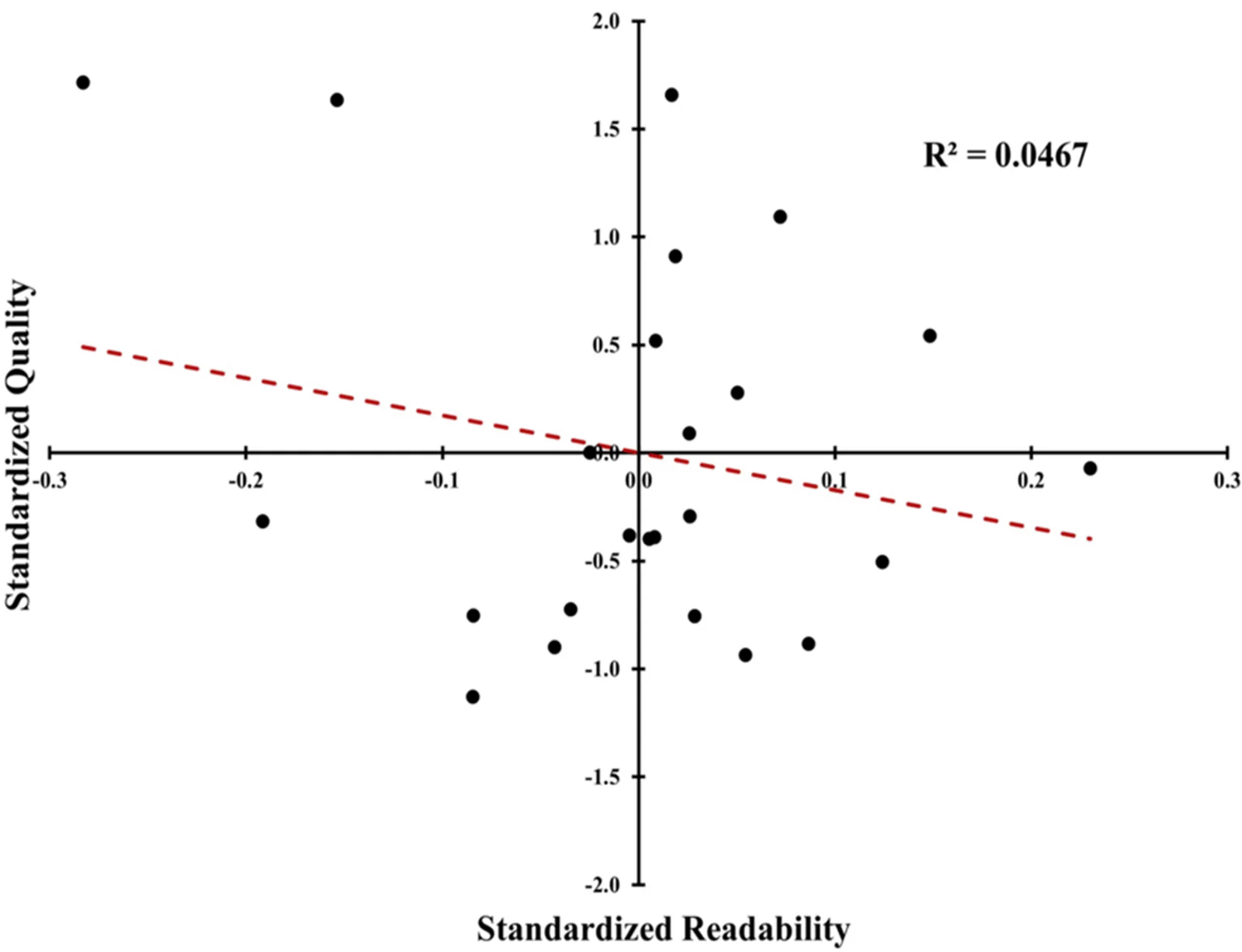
Scatter plot and linear regression analysis of standardized readability versus standardized quality scores The red dashed line indicates the line of best fit (ŷ = −1.732x; R-squared = 0.0467; n = 24).

None of the websites’ categories were below the American Medical Association (AMA) recommendation of a sixth-grade reading level by either the FRES or FKGL metrics [17]. Academic sources most closely approached this threshold with an average reading level of seventh- to eighth-grade. Commercial sources and medical practice sources both had average reading levels of eighth- to ninth-grade. Academic websites had FRES and FKGL scores of 68.46 (eighth- to ninth-grade) and 7.17 (middle school), respectively. Medical practice websites had the next lowest reading level with an FRES average of 68.2 (eighth- to ninth-grade) and an FKGL average of 7.5 (middle school). The most complex average reading level was found on the commercial websites with FRES and FKGL scores of 62.8 (eighth- to ninth-grade) and 8.3 (middle school), respectively (Figure 2). No significant difference was found between either the FRES (p > 0.05) or the FKGL (p > 0.05) scores. The correlation between FKGL and FRES scores was evident when the two values were plotted against each other (Figure 3). The coefficient of determination showed a strong correlation between the two measures (R^2^ = 0.95). There was no significant correlation between readability and quality (R^2^ = 0.0467 and significance of F < 0.32; Figure 4).

## Discussion

Health literacy is an important determinant of patient health outcomes. Low health literacy has been associated with poorer clinical outcomes, increased healthcare spending and utilization, particularly in the form of hospitalizations, as well as higher mortality rates [18]. Reflecting its significance, a validated screening tool for health literacy has even been termed the “new vital sign” [19]. The National Institutes of Health (NIH) defines health literacy as “reading, comprehending, and analyzing information; decoding instructions, symbols, charts, and diagrams; weighing risks and benefits; and, ultimately, making decisions and taking an action” [20]. This definition underscores the foundational role that reading comprehension plays in patient health. When patients are unable to understand the information provided to them, they are significantly limited in their ability to navigate the healthcare system, evaluate options, and make informed decisions. Consequently, general literacy is a critical component of health literacy and population health more broadly.

According to a study done by the Program for International Assessment of Adult Competencies (PIAAC) in 2012 and 2014, 43 million U.S. adults have low literacy skills with a further 63 million U.S. adults having only mid-level literacy skills [21]. Limited general literacy, characterized by a difficulty understanding basic sentences or drawing simple inferences, can directly hinder an individual’s ability to access, interpret, and apply health information [22]. This more specific capacity, known as health literacy, was found to be basic or below basic in 36% of U.S. adults by the National Center for Education Statistics (NCES) [23]. A statement from the American Heart Association [22] connects this inadequate health literacy to a limited knowledge of health conditions [24] and medications [25], poorer overall health status [26], higher healthcare costs [27], and increased likelihood of rehospitalization [28].

High-quality, readable information is furthermore imperative due to the multidisciplinary nature of pediatric skull fracture management. Given the associated sequelae such as hearing loss, cranial nerve injury, and cerebrospinal fluid leaks, these injuries often necessitate collaborative care from a variety of subspecialists, including otolaryngologists, craniofacial surgeons, neurosurgeons, audiologists, and speech-language pathologists. Thus, it is imperative that patients and their guardians have access to understandable information to make this complex intersection of care less daunting and to facilitate informed decision-making in the face of complex comorbidities.

Moreover, because limited health literacy is more prevalent among racial and ethnic minorities, older adults, and individuals with lower educational attainment [23,29], it plays a key role in perpetuating existing health disparities. Considering this, the AMA recommends that written patient materials be composed at a sixth-grade reading level, a standard aimed at making health information accessible to the widest and most vulnerable patient populations [17]. Efforts to enhance health literacy have the potential to reduce disparities in care delivery, improve population-level outcomes, and advance health equity.

Given the pervasive nature of low literacy and its broad impact on general health literacy in the United States [21,23], national health authorities have published quality guidelines and position statements aimed at raising awareness and promoting accountability in the development of patient-facing materials. The AMA provides more stringent recommendations that patient materials be written to no higher than a sixth-grade reading [17]. Using the data from the PIAAC, the U.S. Department of Education NCES suggests that even these recommendations are not sufficient to reach all U.S. adults [21], but these guidelines serve as a base threshold for making health information accessible and equitable.

The data demonstrate that online resources available for pediatric skull fractures do not meet the guidelines for readability. Academic websites trended toward more accessible reading levels, suggesting possible effort on the part of academic institutions to make their materials compliant with the guidelines of the AMA, but this did not achieve statistical significance in terms of readability. A potential explanation for statistically insignificant variance in readability between sample websites is persistent or borrowed verbiage. Words and phrases found on one website often are found repeatedly on related sites. Technical and procedural language inherent to pediatric skull fractures likely also contribute to the higher reading level. It has been shown in other studies that replacing technical words with simpler words will reduce the average reading level [30]. While maintaining accuracy of the published information is vital, deliberate efforts to use more accessible language will increase accessibility without losing the integrity of the work. The inaccessibility of this information suggests it may disproportionately serve those with higher literacy, reinforcing health inequities. Improving readability may be a practical step toward more equitable health communication.

This study also evaluated the quality of the material presented. While there was no difference in readability, there exists a significant difference in QUEST scores, with commercial websites having the highest quality scoring. This difference was not observed when the websites were evaluated using the DISCERN tool. QUEST assigns greater weight to proper citation practices, accountable sourcing of information, the tone of the article, and the disclosure of any conflicts of interest. Although these factors are also considered in DISCERN, they carry less relative weight in that instrument. The higher QUEST scores observed in commercial websites may therefore reflect stronger adherence to these specific attributes. Notably, many academic and medical practice websites appeared to draw from reputable sources but failed to provide explicit citations or direct references, leading to point deductions under the QUEST framework. As a result, lower scores may not indicate inferior content, but rather a lack of transparency in attribution. This distinction underscores a broader concern: while content accuracy may be comparable across website types, the omission of clear sourcing raises issues of accountability and undermines users’ ability to verify information.

Limitations

Current methodologies for evaluating readability and quality of patient-facing information have notable limitations. High rates of site exclusion per project criteria (academic journals, paid access, etc.) severely limit sample size. In addition to weakening statistical power and scope of studies, the exclusion criteria also represent barriers that diminish patients’ ability to access credible and comprehensible healthcare information. Such barriers, use of technical language, foreign methods of information presentation, and financial restrictions, may disproportionately discourage engagement by patients in the healthcare decision-making process, thereby exacerbating existing health literacy disparities. Additionally, this study was restricted to English-language, text-based websites and did not evaluate patient education delivered through videos, social media platforms, or AI-generated information, all of which are increasingly important sources of health information for patients and caregivers. These exclusions may limit the generalizability of our findings to the broader digital health information landscape. Furthermore, the internet is a dynamic, evolving space, and search engine rankings will change with time. This limits the generalizability of our findings to the peri-search period. While improving the readability of materials and adult literacy rates are essential steps, additional efforts must focus on enhancing information accessibility in all its forms to increase the statistical power and reach of health information readability research and ensure effective dissemination to diverse audiences.

## Conclusions

With more patients relying on the internet for their information, it is imperative that we provide adequate and understandable resources to ensure our patients are well informed. This study demonstrates patient-facing, web-based content on pediatric skull fracture is not readily comprehensible for a substantial proportion of the U.S. population. By actively designing materials that meet the needs of diverse audiences, healthcare providers can help reduce the adverse outcomes associated with limited health literacy. Ensuring that all patients are appropriately informed supports better decision-making and mitigates both the causes and consequences of health communication gaps.

## Appendices

Appendix 1 

**Table 3:** A list of the website URLs included for analysis

Number	Website
(1)	https://www.childrenshospital.org/conditions/head-or-brain-injury
(2)	https://www.gosh.nhs.uk/conditions-and-treatments/conditions-we-treat/head-injuries/
(3)	https://www.healthline.com/health/skull-fracture
(4)	https://www.stanfordchildrens.org/en/topic/default?id=head-injury-85-P00785
(5)	https://www.urmc.rochester.edu/childrens-hospital/developmental-disabilities/conditions/head-injury.aspx
(6)	https://www.childbirthinjuries.com/birth-injury/skull-fractures/
(7)	https://kidshealth.org/en/parents/head-injury.html
(8)	https://www.medicalnewstoday.com/articles/322871
(9)	https://www.mountsinai.org/health-library/injury/skull-fracture
(10)	https://www.nicklauschildrens.org/conditions/skull-fracture
(11)	https://www.stanfordchildrens.org/en/topic/default?id=head-injury-in-children-90-P02604
(12)	https://www.healthline.com/health/bone-health/calvarial-fracture
(13)	https://www.childrenscolorado.org/conditions-and-advice/parenting/parenting-articles/head-injuries/
(14)	https://intermountainhealthcare.org/services/pediatrics/services/brain-spine/skull-fracture/
(15)	https://my.clevelandclinic.org/health/diseases/skull-fracture
(16)	https://www.cerebralpalsyguidance.com/birth-injury/infant-skull-fracture/
(17)	https://www.choa.org/medical-services/neurosciences/skull-fractures
(18)	https://www.drugs.com/cg/skull-fracture-in-children.html
(19)	https://www.gosh.nhs.uk/conditions-and-treatments/conditions-we-treat/skull-fracture/
(20)	https://www.hopkinsmedicine.org/health/conditions-and-diseases/head-injury-in-children
(21)	https://www.nationwidechildrens.org/conditions/health-library/head-injury-in-children
(22)	https://www.saintlukeskc.org/health-library/treatment-skull-fracture-child
(23)	https://www.saintlukeskc.org/health-library/understanding-skull-fracture-child
(24)	https://www.uptodate.com/contents/head-injury-in-children-and-adolescents-beyond-the-basics
